# Evaluating the timing of injection laryngoplasty for vocal fold paralysis in an attempt to avoid future type 1 thyroplasty

**DOI:** 10.1186/1916-0216-42-24

**Published:** 2013-03-19

**Authors:** Yazeed Alghonaim, Michael Roskies, Karen Kost, Jonathan Young

**Affiliations:** 1 Department of Otolaryngology – Head & Neck Surgery, McGill University, 687 Pine Ave. West, Montreal, QC, H3A 1A1, Canada

## Abstract

**Objectives:**

To determine whether immediate (less than 3 months from time of nerve injury), early (from 3 to 6 months from time of nerve injury) or late (more than 6 months from time of nerve injury) vocal fold injection influences the long-term outcomes for patients with permanent unilateral vocal fold paralysis.

**Methods:**

A total of 250 patients with documented unilateral vocal fold paralysis were identified in this retrospective chart review. 66 patients met the inclusion criteria, having undergone awake trancervical injection with gelfoam™, collagen, perlane™ or a combination. Patients with documented recovery of vocal fold mobility, or patients with less than one year of follow-up after the onset of paralysis were excluded. Patients were stratified into immediate (<3 months), early (3-6 months) and late (>6 months) groups denoting the time from suspected injury to injection. The need for open surgery as determined by a persistently immobile vocal fold with insufficient glottic closure following injection was the primary outcome.

**Results:**

1 out of 21 (4.8%) in the immediate group, 2 out of 17 (11.8%) in the early group and 20 out of 28 (71.4%) in the late group required type 1 thyroplasty procedures to restore glottic competence. There was significance when comparing late injection to both early and immediate injection (p < 0.001). No statistically significant differences were seen when comparing the number of injections needed to restore glottic competence.

**Conclusions:**

This 10-year longitudinal assessment revealed that early medialization of a permanent paralyzed, abducted vocal fold with a temporary material appears to diminish the likelihood of requiring permanent laryngeal framework surgery.

## Introduction

Vocal fold immobility is a broad term used to describe vocal folds that are restricted secondary to mechanical fixation or neuropathy. Mechanical fixation may result from an arytenoid dislocation, edema or inflammation of the glottis, or neoplastic invasion. Neurogenic immobility may occur with lesions in the motor cortex or compromise of the recurrent laryngeal nerve due to either surgical iatrogenic injury or extra-laryngeal malignancies at any point along its course from the jugular foramen to the carotid sheath, mediastinum, and either around the subclavian artery on the right or the aortic arch on the left, to the tracheoesophageal groove [1]. The resulting glottic insufficiency may lead to dysphonia, aspiration, and shortness of breath. Iatrogenic stretching or transection of the recurrent laryngeal nerve may cause only temporary immobility. However, if there is no recovery, procedures aiming to restore glottic competence include permanent and temporary vocal fold injections (VFI) or laryngeal framework surgery, such as type 1 thyroplasty.

Different injection materials, either permanent (Teflon, PDMS) or temporary (Gelofoam, Cymetra, Restylane, Radiesse) and different approaches (transoral vs. percutaneous) have been used to medialize the paretic vocal fold in order to improve voice and prevent aspiration [2]. Previous approaches to unilateral vocal fold paralysis included waiting several months for spontaneous recovery to be ruled out before proceeding with medialization [3]. Recent evidence, however, suggests that early intervention reduces the need for transcervical reconstruction [4].

The goal was to evaluate the timing of VFI with respect to eventual need for type 1 thyroplasty. In this retrospective chart review, we study the different parameters available to laryngologists for VFI. Specifically, we compare immediate, early and late (>3 months, 3-6 months and >6 months, respectively) injection when using different materials.

## Materials and methods

The study was approved by The Research Ethics Board (REB) of McGill University Health Center (MUHC). A retrospective chart review between Jan 2000 and Sept 2011 identified all adult patients initially presenting at our Voice center and diagnosed during laryngoscopy with UVFP. Of the 250 patients with unilateral vocal fold paralysis, 66 met the inclusion criteria of having undergone injection medialization as initial treatment within 1 year of onset of their paralysis, and these formed the study group.

Patients were stratified into immediate (<3 months), early (3-6 months) and late (>6 months) groups denoting the time from suspected injury/onset of dysphonia to injection. All patients in the study had one or more injections. Hyaluronic acid (perlane™) absorbable gelatin (Gelfoam™) or collagen were injected using a trans-cricothyroid technique. Three senior laryngologists performed the injections on these patients in the same voice lab – all following the same technique. They all injected within each of the immediate, early and late groups. At the time of data collection, 21 patients had injection in <3 months, 17 patients between 3-6 months and 28 had their injections after 6 months. 51 patients had an identifiable cause of paralysis (iatrogenic, malignancy or stab injury), 15 were considered as idiopathic which was confirmed by routine work-up which may have included CT, MRI, barium swallow, thyroid ultrasound, flexible endoscopy and autoimmune workup. Glottic competency was determined based on the patients’ subjective voice quality as well as objectively using the laryngoscope.

## Results

Of the 250 patients with unilateral vocal fold paralysis collected, 66 met the inclusion criteria (31 female, 35 male). The average age of the cohort was 59.5 (range 23 – 84) with no significant differences between the immediate, early and late injection groups. All injections were performed percutaneously in the office setting with local anesthesia and most commonly utilized perlane™ (67%). The descriptive statistics detailing cause of vocal fold paralysis, length of follow-up, timing of injection and outcome (open thyroplasty performed or avoided) are included in Table 1.

**Table 1:** Unilateral vocal fold paralysis patient cohort characteristics

**Age**	**Gender**	**Etiology**	**Length of follow-up months**	**Number of injections**	**Time of thyroplasty type-I**
**Immediate group (n = 21)**					
46	F	Thyroidectomy	48	1	No
53	F	Idiopathic	48	3	No
43	F	Parathyroidectomy	14	2	No
74	M	Neck Ca (chemo/XRT)	30	3	No
66	M	Lung Ca	12	1	No
25	M	Neck stab injury	24	2	No
41	F	Throidectomy	16	1	No
23	M	Gunshot to C1	19	3	No
72	M	Lung Ca	13	2	Yes (13)
53	F	Thyroidectomy	12	3	No
72	M	Lung Ca	12	1	No
82	M	Lung Ca	12	2	No
66	M	Lung Ca	14	2	No
49	F	Thyroidectomy	17	2	No
50	F	Breast Ca	12	1	No
75	M	Lung Ca	12	1	No
39	M	Thyroidectomy	12	1	No
66	M	Lung Ca	14	3	No
70	M	Lung Ca	12	1	No
66	M	Mediastenoscopy	16	3	No
70	M	Lung Ca	12	2	No
**Early group (n = 17)**					
66	M	Lung Ca	12	1	No
77	M	Idiopathic	12	2	Yes (12)
48	F	Thyroidectomy + XRT	24	2	No
70	F	Lung Ca	12	1	No
62	F	Lung Ca	12	1	No
63	F	Lung Ca	24	2	No
66	F	Aortic Surgery	12	2	No
68	M	Lung Ca	30	2	No
59	M	Idiopathic	12	1	No
65	M	Lung Ca	16	2	No
46	F	Mediastenscopy	14	1	Yes (10)
80	M	Idiopathic	20	2	No
60	M	Lung Ca	14	2	No
84	F	Idiopathic	36	2	No
78	M	Mediastinal Ca	12	1	No
70	F	Lung Ca	14	2	No
40	F	NP Ca/XRT	30	2	No
**Late group (n = 28)**					
70	F	NP Ca (XRT)	36	1	Yes (13)
58	F	Thyroidectomy	60	3	No
84	F	Lung Ca	14	1	No
72	F	Lung Ca	14	1	Yes (8)
59	M	Lung Ca	24	1	Yes (14)
64	M	Carotid Surgery	16	1	Yes (20)
67	F	Idiopathic	20	2	Yes (22)
36	F	Idiopathic	60	3	Yes (40)
61	M	Idiopathic	60	3	Yes (26)
40	F	Schwanoma skull base	50	3	Yes (38)
68	M	Lung Ca	36	1	Yes (20)
49	F	Idiopathic	72	7	Yes (50)
71	F	Lung Ca	16	1	No
30	F	Idiopathic	36	2	Yes (30)
60	F	Aortic surgery	20	2	Yes (18)
62	M	Aortic surgery	30	1	Yes (25)
70	M	Idiopathic	48	2	Yes (24)
61	F	Lung Ca	13	1	Yes (12)
60	M	Lung Ca	24	1	Yes (16)
47	M	Idiopathic	36	1	Yes (12)
50	M	Palatectomy/maxillectomy	16	2	Yes (16)
56	M	Idiopathic	32	3	No
42	F	Idiopathic	21	2	Yes (15)
65	F	Lung Ca	14	1	No
49	M	Lung Ca	74	2	Yes (74)
62	M	Lung Ca	28	2	No
40	F	Thyroidectomy	20	1	No
71	M	Idiopathic	25	3	No

**Figure 1:**
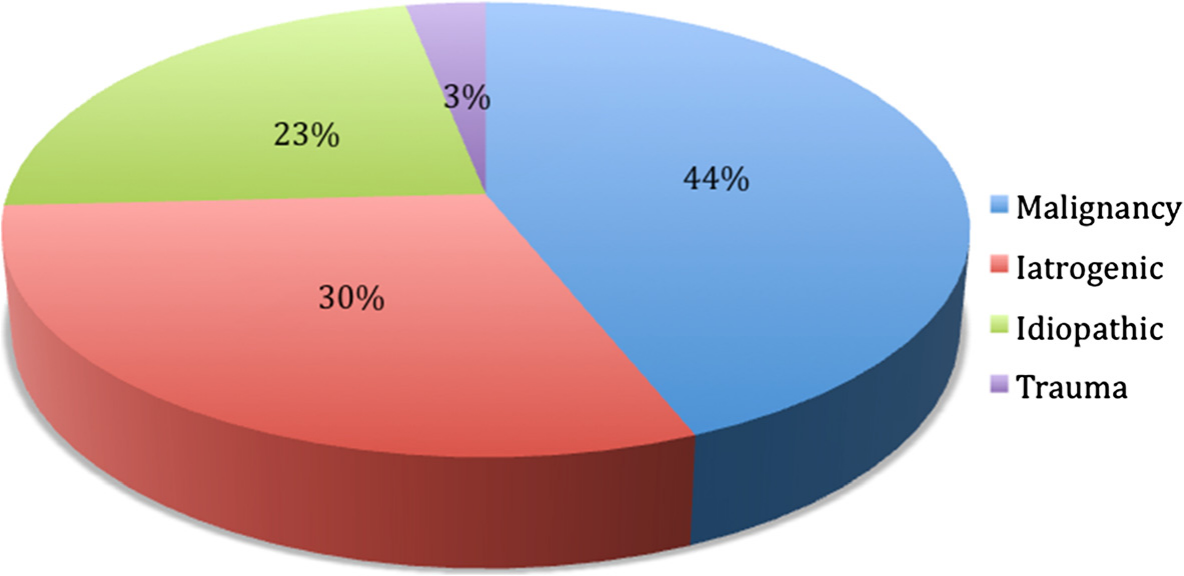
Etiology of unilateral vocal fold immobility.

**Figure 2:**
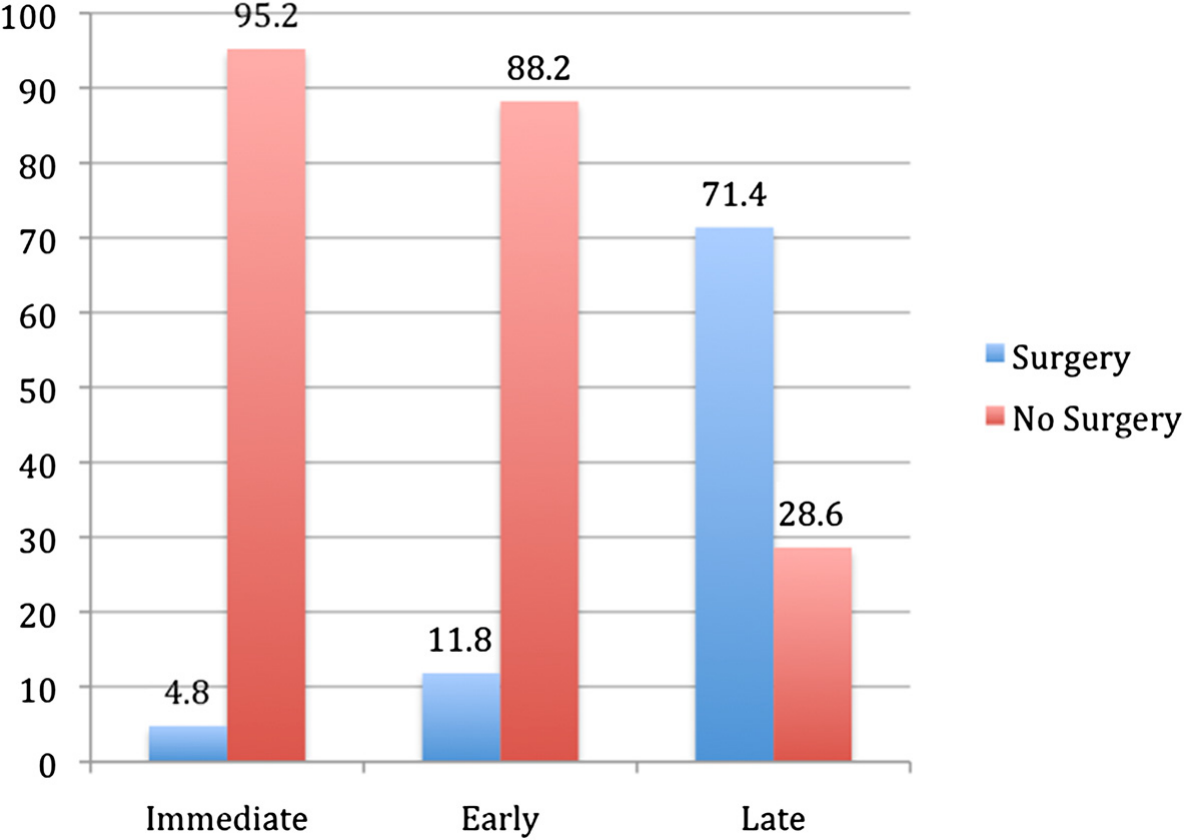
Comparison of need for type-1 thyroplasty at Immediate (<3 months), early (3-6 months) versus late (>6 months) groups.

In total, 29/66 of patients had UVFP secondary to an oncologic etiology with 93% (27/29) a result of lung cancer. 30.3% (20/66) of patients had UVFP from iatrogenic etiologies: 17 post-surgical and 3 from chemotherapy or radiation therapy. The rest of the cohort suffered glottic incompetence from trauma [2] and 15 from idiopathic processes (Figures 1 and 2).

21 patients were stratified into the immediate group (<3 months), 17 patients to the early group (3-6 months) and 28 patients to the late group (>6 months) denoting the time from suspected injury to injection. A mean delay of 42 days for the immediate group, 4.2 months for the early group and 12 months for the late group was observed. The average length of follow up from the onset of dysphonia was 18 and 19 months for immediate (range 12-48 mothns) and early groups (range 12-36 months) respectively and 32.7 months (range 12-74 months) for late group. 62 patients had left and four had right UVFP.

Many of the patients in this study required more than one injection to achieve glottic competency and a satisfactory voice. Thirty-nine (59.1%) required at least two and fifteen (22.7%) required at least three. Two patients required more than 3 injections. Eight patients achieved glottic competency with only one injection from the immediate group compared to six from the early group. Ultimately twenty-three of 66 patients failed to achieve glottic competency and required laryngeal framework surgery and forty-three had documented medialization of the paralyzed vocal fold with a noted improvement in objective and subjective voice quality. Of the twenty-three patients who required laryngeal framework surgery, one was from the immediate group (1/21, 4.8%), two from the early group (2/17, 11.8%) and twenty from the late group (20/28, 71.4%).

Patients who received injections during the immediate 3-month window were 66.7% (95% CI = 47.6 – 85.7) less likely to undergo surgery than those injected after 6 months (P <. 001). Patients who received injections during the early 3 to 6 month window were 59.7% (95% CI = 37 – 82.3) less likely to undergo surgery than those injected after 6 months (P <. 001). Only a 7% difference is seen when comparing the immediate vs. early groups (-10.8 – 24.8) (P >. 05).

## Discussion

The rationale for medializing a paralyzed true vocal fold is to restore glottic competence in order to improve voice quality and prevent aspiration. The optimal time and method of vocal fold paralysis management is controversial. Factors contributing to the controversy include uncertainty regarding the possible return of function, and concern about the irreversibility of some procedures [5]. Initial treatment options for UVFP include temporary vocal fold injection medialization, voice therapy, or observation for spontaneous return of function.

In 1992, Ford and colleagues revolutionized the concept of laryngoplasty with the introduction of a temporary injectable collagen to restore the glottic competence [6]. Since then, several materials for injections have been developed and are typically described as either temporary, long lasting or permanent. Long lasting/permanent injectable materials include autologous fat, calcium hydroxylapatite (Radiesse™), polydimethylsiloxane (PDMS or particulate silicone), and historically, polytef paste (Teflon™) [2]. Temporary injection materials include bovine gelatin (Gelfoam™, Surgifoam™), collagen-based products (Cymetra™, Zyplast™, Cosmoplast/Cosmoderm™), hyaluronic acid (Restylane™, Perlane™, Hyalaform™), and carboxymethylcellulose (Radiesse Voice Gel™) [2].

Understanding the etiology of the paralysis is indeed an essential element of appropriate workup and treatment. Rosenthal and colleagues [1] found that the most common etiology for unilateral immobility is secondary to surgical iatrogenic injury (thyroid and non-thyroid). Aside from surgery, other common causes of vocal fold paralysis include extralaryngeal malignancy, idiopathic causes and trauma. In our cohort, the majority of the patients 43.9% (29/66) had UVFP secondary to a malignant etiology followed by 30.3% (20/66) of patients who had UVFP from iatrogenic etiologies. This distribution might be explained by the referral pattern in our institution and the fact that most of the post thyroidectomy patients were excluded from our sample because they regained their nerve function. The left recurrent laryngeal nerve (94%) was more commonly injured in our study than the right (6%). This is consistent with the prior literatures and can be explained by its longer and more convoluted course.

Previously, it was accepted that waiting several months before intervention would allow time for spontaneous recovery to occur. However, due to its ease of use and low risk and complication rate, early injection laryngoplasty under topical anesthetic provides an excellent therapeutic option for both patients and physician. Awake injection laryngoplasty produces a substantial improvement in voice quality as measured by the Voice-Related Quality of-Life (VRQOL) measure [7]. Moreover, injection laryngoplasty produced improvements in Glottal Function Index (GFI), GRBAS, Functional outcome swallowing scale (FOSS), and maximum phonation time measurements, which confirm the advantage of this technique in improving glottic competency [5]. Bhattacharyya et al. compared early and late vocal fold medialization for vocal fold paralysis following thoracic procedures and found a significantly reduced risk of post-injection pneumonia and length of hospital stay for the early injected group [8]. Friedman et al. recently hypothesized that with early intervention (less than 6 months from time of injury), the implant material allows the vocal fold to be in a more appropriate resting position during the time window of synkinetic reinnervation. It is possible that synkinetic reinnervation permanently maintains a medialized and more favorably positioned vocal fold. Conversely, a non-injected vocal fold which has been assumed a more lateralized (and less favorable) position following synkinetic reinnervation is less likely to be adequately adducted with injection [4]. In fact, there might be a greater degree of benefit to an even earlier acute intervention (i.e., sooner than six months after paralysis) in terms of decreasing the likelihood of requiring a subsequent permanent laryngeal framework procedure [9].

In our study, which defined immediate intervention to be less than three months post paralysis, the percentage of patients requiring open surgery following injections because of inadequate long-term results was 4.8%, which is even lower than (37.5%) what was found by Friedman et al. Only a 7% difference (not statistically significant) is seen when comparing the immediate vs. early groups (-10.8 – 24.8). However, patients who received injections during the immediate or early window were 66.7% and 59.7% (95% CI = 47.6 – 85.7) and (95% CI = 37.0 – 82.3) respectively less likely to undergo surgery than those injected after 6 months (late group). It is a common misconception that vocal fold paralysis is the result of complete muscle denervation. Animal experiments demonstrate that synkinetic reinnervation occurs in more than 65% of all cases of paralysis, which is thought to be comparable to that in humans [10-12].

Many of the patients in this study required more than one injection due to ongoing dysphonia or glottic incompetency. Thirty-nine (59.1%) required at least two injections, including 13 from the immediate, 11 from the early, and 15 from the late groups. Owing to low sample sizes within each group, comparisons between groups are inconclusive. When speaking of recovery, several authors have described return of function in general terms, noting that “all cases recovered in less than twelve months”. However, many documented no further recovery after much shorter intervals [13]. Although delayed recovery as long as 4 years following onset has been very occasionally been noted [14], allowing a one year interval before assuming the paralysis to be permanent and instituting final treatment would seem reasonable. In our sample, all patients who were lost to follow up in less than one year were excluded. The average of follow up from the onset of dysphonia was 18 and 19 months for immediate and early groups respectively, 32.7 months for late group. Finally, patient age has long been recognized as an essential factor in motor nerve regeneration, and many studies have confirmed this finding [15]. Response to the injection and/or thyroplasty was not significantly affected by age in our study.

Our current study has a number of methodologic limitations. First, poor intra-operative documentations of nerve transecting/injury or not, could affect prediction of the final outcome. Second, the data were collected retrospectively on a small sample size and with no EMG study and Speech Language Pathology assessment. Finally, a selection bias exists. Most patients seeking medical therapy after greater than six months of glottic incompetence may have been more likely to accept the need for surgical intervention, compared to the patients that were motivated to undergo early intervention in the form of injection in the office.

## Conclusions

This 10-year longitudinal assessment revealed that patients who received a temporary vocal fold injection for a newly diagnosed vocal fold immobility (less than 6 months) were less likely to undergo permanent medialization laryngoplasty (thyroplasty) compared with those patients who were treated with conservative management alone or had delayed treatment after 6 months. Further studies with a speech language pathology assessment, EMG study and a longer lasting injectables such as radiesse™ for vocal fold paralysis are warranted.

## Competing interests

All authors declare that they have no competing interests.

## Authors’ contributions

YA did the literature review, collected the data, help in analyzing the data, wrote the abstract, introduction, discussion and conclusion. MR wrote the result section, help in data collection, analyze the data. KK: perform the injection and patients follow up, review the final manuscript. JY: perform injection and patients follow up, supervise all the article writing process and finally review the final manuscript. All authors read and approved the final manuscript.
